# Escalating infection control response to the rapidly evolving epidemiology of the coronavirus disease 2019 (COVID-19) due to SARS-CoV-2 in Hong Kong

**DOI:** 10.1017/ice.2020.58

**Published:** 2020-03-05

**Authors:** Vincent C. C. Cheng, Shuk-Ching Wong, Jonathan H. K. Chen, Cyril C. Y. Yip, Vivien W. M. Chuang, Owen T. Y. Tsang, Siddharth Sridhar, Jasper F. W. Chan, Pak-Leung Ho, Kwok-Yung Yuen

**Affiliations:** 1 Department of Microbiology, Queen Mary Hospital, Hong Kong Special Administrative Region, China; 2 Infection Control Team, Queen Mary Hospital, Hong Kong West Cluster, Hong Kong Special Administrative Region, China; 3 Quality & Safety Division (Infection, Emergency, and Contingency), Hospital Authority, Hong Kong Special Administrative Region, China; 4 Infectious Disease Center, Hospital Authority, Hong Kong Special Administrative Region, China; 5 Department of Microbiology, Li Ka Shing Faculty of Medicine, The University of Hong Kong, Hong Kong Special Administrative Region, China

## Abstract

**Objective:**

To describe the infection control preparedness measures undertaken for coronavirus disease (COVID-19) due to SARS-CoV-2 (previously known as 2019 novel coronavirus) in the first 42 days after announcement of a cluster of pneumonia in China, on December 31, 2019 (day 1) in Hong Kong.

**Methods:**

A bundled approach of active and enhanced laboratory surveillance, early airborne infection isolation, rapid molecular diagnostic testing, and contact tracing for healthcare workers (HCWs) with unprotected exposure in the hospitals was implemented. Epidemiological characteristics of confirmed cases, environmental samples, and air samples were collected and analyzed.

**Results:**

From day 1 to day 42, 42 of 1,275 patients (3.3%) fulfilling active (n = 29) and enhanced laboratory surveillance (n = 13) were confirmed to have the SARS-CoV-2 infection. The number of locally acquired case significantly increased from 1 of 13 confirmed cases (7.7%, day 22 to day 32) to 27 of 29 confirmed cases (93.1%, day 33 to day 42; *P* < .001). Among them, 28 patients (66.6%) came from 8 family clusters. Of 413 HCWs caring for these confirmed cases, 11 (2.7%) had unprotected exposure requiring quarantine for 14 days. None of these was infected, and nosocomial transmission of SARS-CoV-2 was not observed. Environmental surveillance was performed in the room of a patient with viral load of 3.3 × 10^6^ copies/mL (pooled nasopharyngeal and throat swabs) and 5.9 × 10^6^ copies/mL (saliva), respectively. SARS-CoV-2 was identified in 1 of 13 environmental samples (7.7%) but not in 8 air samples collected at a distance of 10 cm from the patient’s chin with or without wearing a surgical mask.

**Conclusion:**

Appropriate hospital infection control measures was able to prevent nosocomial transmission of SARS-CoV-2.

A β-coronavirus, SARS-CoV-2, was recognized in a cluster of patients with community-acquired pneumonia in Wuhan, Hubei Province, China, in December 2019.^1^ With the establishment of high-speed rail within China and international travel, this novel coronavirus rapidly disseminated to all provinces of China and 25 countries in the Asia-Pacific region, North America, Europe, and South America within 1 month of its discovery.^2^ Similar to the other β-coronaviruses, such as severe acute respiratory syndrome–associated coronavirus (SARS-CoV) and Middle East respiratory syndrome–associated coronavirus (MERS-CoV), the SARS-CoV-2 is postulated to have originated from bats and to have been transmitted to intermediate hosts before jumping to humans, causing community and nosocomial pneumonia.^3–5^ Before February 11, 2020, the disease caused by this novel coronavirus was temporarily named the 2019 novel coronavirus (2019-nCoV) disease. On February 11, 2020, the World Health Organization renamed the disease the coronavirus disease 2019 (COVID-19), and the virus was classified as SARS-CoV-2 by the International Committee on Taxonomy of Viruses (ICTV). By February 17, 2020, a total of 71,429 people had been infected globally, including 70,635 cases (98.9%) in China. With the addition of 3 patients who died in the Philippines, Japan, and France, 1,772 deaths have been reported in China, with a crude mortality of 2.5%.^6^ Two healthcare workers (HCWs) succumbed as a result of nosocomial acquisition of SARS-CoV-2 in China. In Hong Kong, 8 HCWs died in the outbreak of SARS-CoV in 2003.^3^ Considering our experience with SARS-CoV, it is important to respond to this emerging infectious disease with proactive infection control measures to prevent importation and nosocomial transmission of SARS-CoV-2 in Hong Kong. Here, we report our infection control measures in the first 6 weeks, and the admission of 42 confirmed cases, after the official announcement of a cluster of pneumonia of unknown etiology in Wuhan, Hubei Province, by the National Health Commission of the People’s Republic of China (PRC).

## Methods

### Epidemiology and infection control preparedness for SARS-CoV-2 in Hong Kong

According to the infection control preparedness plan for emerging infectious disease in Hong Kong,^7^ a series of proactive infection control measures were activated by the Hospital Authority, a governing body of all 43 public hospitals responsible for 90% of inpatient service in Hong Kong, immediately after the official announcement of a cluster of pneumonia of unknown etiology in Wuhan, Hubei Province, by the National Health Commission of the PRC on December 31, 2019 (day 1). The key measures included a bundle of infection control measures for early recognition, isolation, notification, and molecular diagnostic testing for all suspected cases.^8^ Active surveillance was performed on patients presenting to the hospital according to a set of clinical and epidemiological criteria, which were adjusted during the evolution of SARS-CoV-2 in China and Hong Kong (Table 1). All suspected cases were isolated in airborne infection isolation rooms (AIIRs) for contact, droplet, and airborne precautions. The Centre for Health Protection, Department of Health, and the Hospital Authority were notified of suspected cases. Enhanced laboratory surveillance was also conducted to widen the scope of screening (Table 1). Patients received AIIR care when available; otherwise, patients received care in a ward with 1-m spacing between patients.

**Table 1.** Surveillance Program for Early Recognition of Patients With Severe Acute Respiratory Syndrome Coronavirus 2 (SARS-CoV-2) in Hong Kong

Active Surveillance for Imported Cases^a^ (from 31 December 2019, day 1)^b^		
A.	Clinical criteria (evolving with time)	Remark
1.	Presented with fever and acute respiratory illness, or pneumonia (from day 1 to day 23)	Prepare for the importation of an index patient to Hong Kong
2.	Presented with fever or acute respiratory illness or pneumonia (with effect from day 24)	
B.	Epidemiological criteria (evolving with time)^c^	
1.	History of travel to Wuhan, Hubei Province, within 14 d before onset of symptoms, irrespective of any exposure to wet market or seafood market (from day 1 to day 16)	Prepare for the importation of an index patient to Hong Kong
2.	Patient had any of the following within 14 d prior to the onset of symptoms: (a) Visited Wuhan (regardless of whether the individual had visited wet markets or seafood markets there) (b) Visited a medical hospital in mainland China (c) Had close contact with a confirmed case of the novel coronavirus while that patient was symptomatic (from day 17 to day 20)	Response to the evolving epidemic with increasing number of confirmed cases in Wuhan
3.	Patient met any of the following criteria within 14 d prior to the onset of symptoms: (a) Visited Hubei Province (regardless of whether the individual had visited wet markets or seafood markets there) (b) Visited a medical hospital in mainland China (c) Had close contact with a confirmed case of the novel coronavirus while that patient was symptomatic (from day 17 to day 20) (with effect from day 21)	Response to spread of SARS-CoV-2 beyond Wuhan

Enhanced Laboratory Surveillance to Identified Imported and Locally Acquired Cases (starting January 13, 2020, day 14, and evolving witd time)^d^		
C.	Patient with pneumonia	Remark
1	(a) Unknown causes (not responding to treatment in 3 d) or (b) Requiring ICU care or occurring in clusters or (c) A healthcare worker (from day 14 to day 21)	Response to the evolving epidemic with increasing number of confirmed cases in Wuhan, and other parts of China
2.	Point C1 or any inpatient with pneumonia and history of travel to mainland China within 14 d before onset of symptoms (from day 22)	
3.	Community-acquired pneumonia without history of travel (from day 32)	Response to the confirmed local case in Hong Kong

a Application for Accidental and Emergency Department (AED), outpatient clinics, and day centers to prevent the importation of a patient with SARS-CoV-2. Patients fulfilling clinical and epidemiological criteria are to be isolated in airborne infection isolation room, reported to the Centre for Health Protection, Department of Health, and tested for SARS-CoV-2 by reverse transcription polymerase chain reaction (RT-PCR).

b Day 1 is denoted as the day of official announcement of a cluster of pneumonia in Wuhan, Hubei Province, by the PRC National Health Commission.

c Epidemiological criteria have been updating according to the spread of SARS-CoV-2.

d Serving as safety net to detect infected patient without a clear epidemiological exposure.

Upper respiratory specimens (ie, nasopharyngeal aspirates, or flocked swabs, and throat swabs) were collected for all cases under active and enhanced laboratory surveillance, whereas lower respiratory specimens (ie, sputum, endotracheal aspirates, or bronchoalveolar lavage) were collected for rapid molecular diagnostic testing if it was available. The molecular diagnostic testing was simultaneously performed by the Public Health Laboratory Service, the Centre for Health Protection, and Queen Mary Hospital, The University of Hong Kong, during the initial phase of the preparedness measures, with a turnaround time of 4–8 hours, depending on the number of specimens per batch. With the increasing number of tests, molecular diagnostic testing has been performed by 7 microbiology laboratories in 7 regional hospitals, including Queen Mary Hospital in Hong Kong, since February 1, 2020 (day 33).

The epidemiology of confirmed cases was analyzed. Imported cases were defined as patients with history of travel to the affected areas 14 days before symptom onset. A local case was defined as a patient with no history of travel to the affected areas 14 days before onset of symptoms. Enhanced infection control measures with clear illustrations regarding the choice of personal protective equipment (PPE) were enforced (Table 2). Regular, open-staff forums were held, along with face-to-face education sessions, to provide “right-on-time” infection control updates and to address staff concerns. Practical training sessions using PPE were performed by the hospital infection control team. Hand hygiene compliance assessments were conducted regularly in our hospitals.

**Table 2.** Enhanced Infection Control Measures to Prevent Nosocomial Transmission of Severe Acute Respiratory Syndrome Coronavirus 2 (SARS-CoV-2) in Hong Kong

Control Measure	Caring for Suspected or Confirmed Cases of SARS-CoV-2^a^	Triage Station^b^	Aerosol Generating Procedures^c^	Other Wards or Patient Areas^d^	Other Area With No Direct Patient Contact
Hand hygiene	Required	Required	Required	Required	Required
Choice of mask	N95 respirator	N95 respirator^e^	N95 respirator	Surgical mask	Surgical mask
Isolation gown	AAMI level 3^f^	AAMI level 1 or 3^f^	AAMI level 1 or 3^f^	Standard precautions +/− transmission-based precautions	Not required
Disposable gloves	Required	Risk assessment	Required		Not required
Eye protection	Goggles, face shield	Eye visor, goggles, face shield	Goggles, face shield		Not required
Hair cover	Optional	Optional	Optional		Not required

a Suspected or confirmed cases of SARS-CoV-2 receive care in airborne infection isolation rooms.

b Including triage stations of emergency rooms and outpatient clinics.

c Aerosol generating procedures included endotracheal intubation, cardiopulmonary resuscitation, bronchoscopy, and open suction of respiratory tract, sputum induction, use of nebulizer therapy, noninvasive positive pressure ventilation, and high-frequency oscillatory ventilation.

d Including outpatient clinics, radiological facilities, physiotherapy, occupation therapy, and day centers.

e Surgical mask could be used as an alternative based on risk assessment.

f AMMI, Association for the Advancement of Medical Instrumentation PB70:2003 is to define the liquid barrier performance and classification of protective apparel and drapes intended for use in healthcare facilities (https://www.fda.gov/medical-devices/personal-protective-equipment-infection-control/medical-gowns). AAMI level 1 isolation gowns are used when small amounts of fluid exposure are anticipated, and AAMI level 3 isolation gowns are used when large amounts of fluid exposure are anticipated.

### Investigating possible nosocomial transmission of SARS-CoV-2

Upon laboratory confirmation of a patient with SARS-CoV-2, the infection control team immediately followed up to identify HCWs and patients with unprotected exposure. This procedure basically followed the contact-tracing protocol of avian influenza A H7N9 in Hong Kong.^9^ Briefly, close contact refers to those with unprotected exposure, defined as HCWs who had provided care for a case patient with inappropriate PPE and patients who had stayed within the same cubicle of the index case regardless of the duration of exposure. Persons deemed to have had close contact with unprotected exposure were required to remain in quarantine for 14 days since last exposure, followed by medical surveillance for 14 days after completion of the quarantine period. During medical surveillance, these people were advised to wear surgical masks in the hospital and the community.

### Laboratory diagnosis of SARS-CoV-2

Clinical specimens including nasopharyngeal aspirates, nasopharyngeal swabs, throat swabs, saliva, sputum, endotracheal aspirates, or bronchoalveolar lavage were first mixed into 2 mL viral transport medium (VTM), and 250-μL samples were subjected to nucleic acid extraction by the eMAG extraction system (bioMérieux, Marcy-l’Étoile France), with an elution volume of 55 μL.

Before the identification of SARS-CoV-2, a pan-coronavirus polymerase chain reaction (PCR) assay with modification to detect 23 coronaviruses known to be present in human, animals, and bats was used.^8,10^ Subsequently, real-time PCR targeting the E gene of the SARS-CoV-2/SARS-like coronavirus was performed using the LightMix Modular SARS and Wuhan CoV E-gene mix (TIB Molbiol, Berlin, Germany) and the LightCycler Multiplex RNA Virus Master Kit (Roche Diagnostics, Mannheim, Germany). Briefly, a 20-μL reaction contained 10 μL RNA templates, 4 μL 5 × RT-qPCR reaction buffer, 0.5 μL LightMix reagent mix, 0.1 μL 200 × RT enzyme, and 5.4 μL nuclease-free H_2_O. Thermal cycling was performed at 55°C for 5 minutes for reverse transcription, followed by 95°C for 5 minutes, then 45 cycles of 95°C for 5 seconds, 60°C for 15 seconds, and 72°C for 15 seconds on the LightCycler 480 II system (Roche Diagnostics, Mannheim, Germany). The SARS-CoV-2 RNA loads in patient and environmental samples were determined using a real-time RT-PCR assay developed in house to target the SARS-CoV-2 RdRp gene^11^.

### Environmental surveillance for SARS-CoV-2

Air samples for SARS-CoV-2 RNA were collected for the first confirmed case in Hong Kong by an air sampler, SAS Super ISO 180 model 86834 (VWR International PBI Srl, Milan, Italy) with modification as previously described.^12,13^ Briefly, the air sampler was perpendicularly positioned at a distance of 10 cm at the level of patient’s chin, and 1,000 L air at a rate of 180 L per minute was collected for each culture plate containing 3 mL of VTM. The patient was instructed to perform 4 different maneuvers (ie, normal breathing, deep breathing, speaking “1, 2, 3” continuously, and coughing continuously) while putting on and putting off the surgical mask, which complied with the ASTM F2100 level 1 standard. The VTM was transferred to the laboratory within 2 hours and was subjected to RT-PCR for the detection of SARS-CoV-2.

Swab samples (Oxoid Transport Swabs, Copan Italia, Italy) from the patient’s environment including bench, bedside rail, locker, bed table, alcohol dispenser, and window bench, before and after collection of air samples, were collected and tested for SARS-CoV-2 using RT-PCR. Briefly, swab samples covering a mean surface area of 9 cm^2^ (3 cm × 3 cm) were submerged in 2 mL VTM. The VTM was further centrifuged at 13,000 ×*g* for 1 minute, and 1 mL of the supernatant was used for nucleic acid extraction. A nasopharyngeal flocked swab, throat swab, and saliva of this patient were collected on the day of environmental surveillance and were subjected to viral load assay.

This study was approved by the Institutional Review Board of The University of Hong Kong/Hospital Authority Hong Kong West Hospital Cluster.

### Statistical analysis

We used the Fisher exact test to compare independent categorical variables between groups. All reported *P* values were 2-sided. *P* < .05 was considered statistically significant. Computation was performed using the SPSS version 15.0 software for Windows (IBM, Armonk, NY).

## Results

### Epidemiology and infection control preparedness for SARS-CoV-2 in Hong Kong

**Fig. 1.**
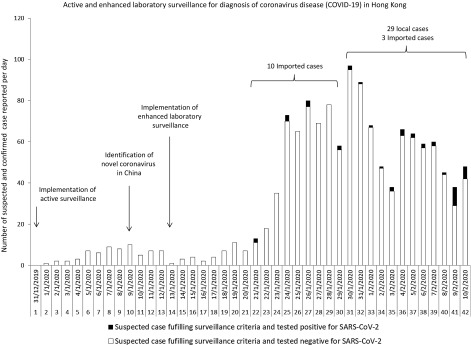
Active and enhanced laboratory surveillance for diagnosis of SARS-CoV-2 in Hong Kong. Both calendar date and day after official announcement of a cluster of pneumonia in Wuhan, Hubei Province, by the PRC National Health Commission on December 31, 2019, are shown. From day 1 to day 20, pan-coronavirus PCR with modification to detect 23 coronaviruses known to be present in human, animals, and bats was used. From day 21 onward, real-time PCR targeting the E gene of the SARS-CoV-2/SARS-like coronavirus was performed using the LightMix Modular SARS and Wuhan CoV E-gene mix (TIB Molbiol, Berlin, Germany) and the LightCycler Multiplex RNA Virus Master Kit (Roche Diagnostics, Mannheim, Germany).

Up to February 10, 2020 (day 42 after official announcement of a cluster of pneumonia of unknown etiology in Wuhan, Hubei Province), 1,275 patients fulfilled the clinical and epidemiological criteria for active and enhanced surveillance upon presentation to our public hospitals, of whom 42 of these 1,275 patients (3.3%) were confirmed to be a cases of COVID-19 in Hong Kong (Fig. 1). Among these patients, 20 were male and 22 were female, with a median age of 59 years (range, 22–91 years); 9 of these patients were residents of mainland China (7 from Wuhan, 1 from Shenzhen, and 1 from Zhuhai), who had arrived by high-speed train (n = 6), by flight (n = 2), and by bus (n = 1). The remaining 33 patients were Hong Kong residents, 5 of whom had a history of travel to mainland China in the 14 days before the onset of symptoms. Exposure to a wet or seafood market was reported by 2 patients. The first patient was confirmed on January 21, 2020 (day 22). From day 22 to day 32, only 1 of 13 confirmed cases (7.7%) was locally acquired. The number of locally acquired cases significantly increased to 27 of 29 confirmed cases (93.1%) from day 33 to day 42 (*P* < .001, Fisher exact test). These cases occurred in 8 family cluster involving 28 patients. One patient (2.4%) died and 4 patients (9.5%) remained in critical condition requiring mechanical ventilation as at day 42.

### Investigating possible nosocomial transmission of SARS-CoV-2

Upon epidemiological investigation of 42 confirmed cases, 36 patients were directly admitted to AIIR, and 6 patients initially received care in non-AIIR facilities. Of the 413 HCWs caring for these patients before confirmation of SARS-CoV-2, 11 HCWs (2.7%) had been in close contact with unprotected exposure and required quarantine for 14 days. None of them was infected with SARS-CoV-2 by the end of the quarantine. Nosocomial transmission was not observed in these hospitalized patients.

### Environmental surveillance for SARS-CoV-2

The viral loads of the first confirmed case were 3.3 × 10^6^ copies per mL in the pooled nasopharyngeal and throat swabs and 5.9 × 10^6^ copies per mL in saliva on the day of air sampling. The air samples collected while the patient performed 4 different maneuvers (ie, normal breathing, deep breathing, speaking “1, 2, 3” continuously, and coughing continuously) while putting on and putting off the surgical mask were all undetectable for SARS-CoV-2 RNA. The viral load of the window bench was 6.5 × 10^2^ copies per mL of VTM before the collection of air samples, but the other environmental samples collected before and after the air sampling had no detectable SARS-CoV-2 RNA. The environmental and air samples were collected by an experienced infection control nurse wearing full PPE including N95 respirator, face shield, cap, gloves, and gown. This nurse was in close contact with the confirmed case for a total of 63 minutes. She completed 14 days of medical surveillance without developing fever or respiratory symptoms.

## Discussion

The emergence of COVID-19, the SARS-CoV-2–associated pneumonia, poses a global threat and challenges to communities as well as healthcare systems. In response to this unprecedented outbreak, which has already produced a higher number of infected cases and mortality within the first 6 weeks of its declaration than the entire outbreak of SARS-CoV in 2003,^2,3^ a rapid infection control response is essential to contain and mitigate the risk of nosocomial transmission and outbreak. In the SARS-CoV outbreak, almost 60% of nosocomial acquisition of SARS-CoV occurred among HCWs^4^; therefore, it is critically important to implement proactive infection control measures among HCWs, and these measures must be planned in advance. In Hong Kong, a cosmopolitan city of 1,104 km^2^ with a population of 7.45 million in southern China, we are at a high risk of importation of infected cases from mainland China. Therefore, we progressively stepped up our infection control measures by widening the clinical and epidemiological criteria of surveillance for early recognition and isolation of index cases according to the evolution of the epidemic. In particular, having visited a hospital in mainland China was introduced as an epidemiological criterion for surveillance on day 17 of our infection control preparedness measures, even though COVID-19 was confined to Wuhan, Hubei Province, until day 20.^2^ The criteria of previous hospital visit was included because it had previously been determined to be a risk factor for SARS acquisition in China.^14^ Under the surveillance program, of 42 cases of COVID-19 were identified in Hong Kong, 36 were immediately isolated in AIIR upon admission. During the SARS outbreak, the median time between index patient admission and patient isolation was 4.5 days (1–13 days), according to a review of literature.^4^

At the same time, we enhanced the infection control measures by implementing standard, contact, droplet, and airborne precautions for suspected or confirmed cases. We stepped up the use of PPE among HCWs performing aerosol-generating procedures (AGPs), even when caring for patients without clinical features and epidemiological exposure risk in the general wards. Performance of AGPs such as endotracheal intubation, open suctioning, and use of high-flow oxygen was a risk factor for nosocomial transmission of SARS-CoV among HCWs.^15^ In addition, provision of surgical masks to all HCWs, patients, and visitors in clinical areas was implemented on day 5. Although wearing a surgical mask alone was not clearly associated with protection from acquisition of SARS-CoV, wearing a surgical mask by either HCWs or patients reduces the risk of nosocomial transmission of pandemic influenza.^16,17^ The combination of hand hygiene with face masks shows statistically significant efficacy against laboratory-confirmed influenza in the community, as illustrated in a systematic review and meta-analysis.^18^ Hand hygiene among HCWs and patients was promoted and enforced during the COVID-19 epidemic.^19,20^ With this bundle of infection prevention measures, we were able to maintain zero nosocomial transmission of SARS-CoV-2 after the importation of first confirmed case on day 22 in Hong Kong.

The mode of transmission of SARS-CoV-2 will undoubtedly be investigated further. Opportunistic airborne transmission was implicated in SARS-CoV.^21^ The World Health Organization recommends the use of airborne precautions whenever applicable in addition to standard, contact, and droplet precautions.^22^ To investigate this connection, we conducted a pilot experiment to examine the exhaled air of a confirmed patient with a moderate level of viral load in respiratory specimens, with or without wearing a surgical mask in the AIIR. Notably, the RNA of SARS-CoV-2 was undetectable in the air samples but was present in an environmental sample. We cannot make a definite conclusion based on the analysis of a single patient; however, our finding may help to reassure our staff that exhaled air may be rapidly diluted inside an AIIR with 12 air exchanges per hour, or that the SARS-CoV-2 may not be predominantly transmitted by airborne route. The presence of environmental contamination by SARS-CoV-2 highlights the importance of transmission via direct or indirect contact. SARS-CoV retained its viability on a smooth surface for >5 days at temperatures of 22–25°C and relative humidity of 40%–50%.^23^

Transmission within families remained a concern because 66% of confirmed cases diagnosed in Hong Kong were spread among family members. One family cluster comprised 11 cases, most probably caused by viral transmission during their gathering for hot pot, in which the use of utensils and chopsticks contaminated by saliva may occur. Saliva was shown to be positive for SARS-CoV-2 in 11 of 12 patients at a median of 3.3 × 10^6^ copies per mL at the time of presentation.^24^ In this family cluster, an asymptomatic patient was retrospectively diagnosed, a 91-year-old lady. Along with our recent report of an asymptomatic case in a pediatric patient,^25^ we observe that asymptomatic infection can occur over a wide age range. The transmissibility of infection among asymptomatic patients deserves further investigation.

With the implementation of active and enhanced surveillance with progressively wider screening criteria during the evolution of this epidemic, we have recognized most of the confirmed cases upon hospitalization, and we have achieved zero nosocomial transmission in HCWs and patients within the first 6 weeks. However, our surveillance program may be challenged by patients with mild symptoms. In early publications, fever and cough were reported in 87% and 80% of patients, respectively, at the time of presentation.^1,25–30^ With the presence of a locally acquired case, epidemiological criteria may no longer be useful for admission screening. Vigilance in hand hygiene practice, wearing of surgical masks in the hospital, and appropriate use of PPE in patient care, especially performing AGPs, are the key infection control measures to prevent nosocomial transmission of SARS-CoV-2, even before the availability of effective antiviral agents and vaccine.

## References

[1] Zhu N , Zhang D , Wang W , et al. A novel coronavirus from patients with pneumonia in China, 2019. N Engl J Med 2020;382:727–733. 10.1056/NEJMoa2001017 PMC7092803 31978945

[2] Novel Coronavirus (2019-nCoV) situation report-1. World Health Organization website. https://www.who.int/docs/default-source/coronaviruse/situation-reports/20200121-sitrep-1-2019-ncov.pdf?sfvrsn=20a99c10_4 Published January 21, 2020. Accessed February 10, 2020.

[3] Cheng VC , Lau SK , Woo PC , Yuen KY. Severe acute respiratory syndrome coronavirus as an agent of emerging and reemerging infection. Clin Microbiol Rev 2007;20:660–694. 17934078 10.1128/CMR.00023-07 PMC2176051

[4] Cheng VC , Chan JF , To KK , Yuen KY. Clinical management and infection control of SARS: lessons learned. Antiviral Res 2013;100:407–419. 23994190 10.1016/j.antiviral.2013.08.016 PMC7132413

[5] Chan JF , Lau SK , To KK , Cheng VC , Woo PC , Yuen KY. Middle East respiratory syndrome coronavirus: another zoonotic betacoronavirus causing SARS-like disease. Clin Microbiol Rev 2015;28:465–522. 25810418 10.1128/CMR.00102-14 PMC4402954

[6] Coronavirus disease 2019 (COVID-19) situation report-28. World Health Organization website. https://www.who.int/docs/default-source/coronaviruse/situation-reports/20200217-sitrep-28-covid-19.pdf?sfvrsn=a19cf2ad_2 Published February 17, 2020. Accessed February 19, 2020.

[7] Wong ATY , Chen H , Liu SH , et al. From SARS to avian influenza preparedness in Hong Kong. Clin Infect Dis 2017;64:S98–S104. 28475794 10.1093/cid/cix123

[8] Cheng VCC , Wong SC , To KKW , Ho PL , Yuen KY. Preparedness and proactive infection control measures against the emerging novel coronavirus in China. J Hosp Infect 2020. pii: S0195-6701(20)30034-7. doi: 10.1016/j.jhin.2020.01.010. PMC7134450 31962139

[9] Cheng VC , Tai JW , Lee WM , et al. Infection control preparedness for human infection with influenza A H7N9 in Hong Kong. Infect Control Hosp Epidemiol 2015;36:87–92. 25627766 10.1017/ice.2014.2

[10] Yip CC , Lam CS , Luk HK , et al. A six-year descriptive epidemiological study of human coronavirus infections in hospitalized patients in Hong Kong. Virol Sin 2016;31:41–48. 26920709 10.1007/s12250-016-3714-8 PMC7090542

[11] Chan JF , Yip CC , To KK , et al. Improved molecular diagnosis of COVID-19 by the novel, highly sensitive and specific COVID-19-RdRp/Hel real-time reverse transcription-polymerase chain reaction assay validated *in vitro* and with clinical specimens. J Clin Microbiol 2020. pii: JCM.00310-20. doi: 10.1128/JCM.00310-20. PMC7180250 32132196

[12] Cheng VCC , Wong SC , Chiu KHY , Yip CCY , Wong SCY , Yuen KY. Detection of norovirus in air samples in a non-vomiting patient: implications of testing saliva for norovirus in an immunocompromised host. J Hosp Infect 2019;103:357–358. 31352056 10.1016/j.jhin.2019.07.011

[13] Cheng VCC , Wong SC , Wong SCY , et al. Measles outbreak from Hong Kong International Airport to the hospital due to secondary vaccine failure in healthcare workers. Infect Control Hosp Epidemiol 2019;40:1407–1415. 31587686 10.1017/ice.2019.278

[14] Wu J , Xu F , Zhou W , et al. Risk factors for SARS among persons without known contact with SARS patients, Beijing, China. Emerg Infect Dis 2004;10:210–216. 15030685 10.3201/eid1002.030730 PMC3322931

[15] Loeb M , McGeer A , Henry B , et al. SARS among critical care nurses, Toronto. Emerg Infect Dis 2004;10:251–255. 15030692 10.3201/eid1002.030838 PMC3322898

[16] Cheng VC , Tai JW , Wong LM , et al. Prevention of nosocomial transmission of swine-origin pandemic influenza virus A/H1N1 by infection control bundle. J Hosp Infect 2010;74:271–277. 20061056 10.1016/j.jhin.2009.09.009 PMC7118838

[17] Cheng VC , To KK , Tse H , Hung IF , Yuen KY. Two years after pandemic influenza A/2009/H1N1: what have we learned? Clin Microbiol Rev 2012;25:223–263. 22491771 10.1128/CMR.05012-11 PMC3346300

[18] Wong VW , Cowling BJ , Aiello AE. Hand hygiene and risk of influenza virus infections in the community: a systematic review and meta-analysis. Epidemiol Infect 2014;142:922–932. 24572643 10.1017/S095026881400003X PMC4891197

[19] Cheng VC , Tai JW , Li WS , et al. Implementation of directly observed patient hand hygiene for hospitalized patients by hand hygiene ambassadors in Hong Kong. Am J Infect Control 2016;44:621–624. 26777285 10.1016/j.ajic.2015.11.024

[20] Cheng VCC , Wong SC , Wong SCY , Yuen KY. Directly observed hand hygiene—from healthcare workers to patients. J Hosp Infect 2019;101:380–382. 30496764 10.1016/j.jhin.2018.11.016

[21] Roy CJ , Milton DK. Airborne transmission of communicable infection—the elusive pathway. N Engl J Med 2004;350:1710–1712. 15102996 10.1056/NEJMp048051

[22] Infection prevention and control during health care when novel coronavirus (nCoV) infection is suspected. World Health Organization website. https://www.who.int/publications-detail/infection-prevention-and-control-during-health-care-when-novel-coronavirus-(ncov)-infection-is-suspected-20200125. Published January 25, 2020. Accessed February 10, 2020.

[23] Chan KH , Peiris JS , Lam SY , Poon LL , Yuen KY , Seto WH. The effects of temperature and relative humidity on the viability of the SARS coronavirus. Adv Virol 2011;2011:734690. 22312351 10.1155/2011/734690 PMC3265313

[24] To KKW , Tsang OTY YC , Chan KH , et al. Consistent detection of 2019 novel coronavirus in saliva. Clin Infect Dis 2020. doi: 10.1093/cid/ciaa149. PMC7108139 32047895

[25] Chan JF , Yuan S , Kok KH , et al. A familial cluster of pneumonia associated with the 2019 novel coronavirus indicating person-to-person transmission: a study of a family cluster. Lancet 2020;395:514–523. 31986261 10.1016/S0140-6736(20)30154-9 PMC7159286

[26] Huang C , Wang Y , Li X , et al. Clinical features of patients infected with 2019 novel coronavirus in Wuhan, China. Lancet 2020;395:497–506. 31986264 10.1016/S0140-6736(20)30183-5 PMC7159299

[27] Phan LT , Nguyen TV , Luong QC , et al. Importation and human-to-human transmission of a novel coronavirus in Vietnam. N Engl J Med 2020. doi: 10.1056/NEJMc2001272. PMC7121428 31991079

[28] Ren LL , Wang YM , Wu ZQ , et al. Identification of a novel coronavirus causing severe pneumonia in human: a descriptive study. Chin Med J (Engl) 2020. PMID: 32004165. doi: 10.1097/CM9.0000000000000722. PMC7147275 32004165

[29] Holshue ML , DeBolt C , Lindquist S , et al. First case of 2019 novel coronavirus in the United States. N Engl J Med 2020. doi: 10.1056/NEJMoa2001191. PMC7092802 32004427

[30] Chen N , Zhou M , Dong X , et al. Epidemiological and clinical characteristics of 99 cases of 2019 novel coronavirus pneumonia in Wuhan, China: a descriptive study. Lancet 2020;395:507–513. 32007143 10.1016/S0140-6736(20)30211-7 PMC7135076

